# A Simple Method to Detect SARS-CoV-2 in Wastewater at Low Virus Concentration

**DOI:** 10.1155/2022/4867626

**Published:** 2022-02-22

**Authors:** Supranee Thongpradit, Somsak Prasongtanakij, Supanart Srisala, Yothin Kumsang, Suwannee Chanprasertyothin, Pairoj Boonkongchuen, Dhanesh Pitidhammabhorn, Parnrudee Manomaipiboon, Peeraya Somchaiyanon, Siriwan Chandanachulaka, Taiyatach Hirunrueng, Boonsong Ongphiphadhanakul

**Affiliations:** ^1^Research Center, Faculty of Medicine Ramathibodi Hospital, Mahidol University, Salaya, Thailand; ^2^Chakri Naruebodindra Medical Institute, Faculty of Medicine Ramathibodi Hospital, Mahidol University, Salaya, Samut Prakan, Thailand; ^3^Health Department, Bangkok Metropolitan Administration, Bangkok, Thailand; ^4^Department of Health, Ministry of Public Health, Nonthaburi, Thailand; ^5^Department of Medicine, Faculty of Medicine Ramathibodi Hospital, Mahidol University, Salaya, Thailand

## Abstract

**Background:**

Since its initial appearance in December 2019, coronavirus disease 2019 (COVID-19), caused by severe acute respiratory syndrome coronavirus 2 (SARS-CoV-2), has spread globally. Wastewater surveillance has been demonstrated as capable of identifying infection clusters early. The purpose of this study was to investigate a quick and simple method to detect SARS-CoV-2 in wastewater in Thailand during the early stages of the second outbreak wave when the prevalence of the disease and the virus concentration in wastewater were low.

**Methods:**

Wastewater samples were collected from a hospital caring for patients with COVID-19 and from 35 markets, two of which were associated with recently reported COVID-19 cases. Then, samples were concentrated by membrane filtering prior to SARS-CoV-2 detection by RT-qPCR.

**Results:**

SARS-CoV-2 RNA was detected in the wastewater samples from the hospital; the Ct values for the N, ORF1ab, and S genes progressively increased as the number of patients admitted to the treatment floor decreased. Notably, the ORF1ab and S genes were still detectable in wastewater even when only one patient with COVID-19 remained at the hospital. SARS-CoV-2 RNA was detected in the wastewater samples from fresh market where COVID-19 cases were reported.

**Conclusions:**

Our findings suggest that wastewater surveillance for SARS-CoV-2 is sensitive and can detect the virus even in places with a high ambient temperature and relatively low prevalence of COVID-19.

## 1. Background

Wastewater surveillance for SARS-CoV-2 has been demonstrated to be a feasible and sensitive method for assessing the prevalence and monitoring the transmission of SARS-CoV-2 in various countries and situations during the ongoing COVID-19 pandemic. It was initially shown that SARS-CoV-2 can be detected in both untreated [1–5] and treated wastewater [6] in several countries. Similar results from additional studies in multiple geographical areas have led to the United States Center for Disease Control to recommend wastewater surveillance for SARS-CoV-2 [7].

The spread of COVID-19 appears to be lessened by warm and wet weather [3], and there is a negative correlation between temperature and COVID-19 prevalence across countries. Notably, moderate temperature increases to 34°C can disrupt the structure of SARS-CoV-2, while humidity has very little impact [8]. For the related virus, severe acute respiratory syndrome (SARS), UV irradiation of >90 *μ*w/cm^3^ to the culture medium for 60 min can completely impair viral infectivity [9]. Furthermore, UV radiation has been shown to have a significant association with the incidence of COVID-19, which may help to flatten the epidemic [10]. To date, most studies examining the utilization of wastewater surveillance for SARS-CoV-2 were performed in countries with cold weather and/or a high COVID-19 prevalence. For tropical countries, at least in India during 2020, when the average temperature was 25.78 degrees Celsius (°C) [11], SARS-CoV-2 could be molecularly detected in influent wastewater from a water treatment plant. However, at the time of that study, the prevalence of SARS-CoV-2 in India was very high [12]. Thailand is located in the tropical area where the climate is warm to hot year-round with average temperatures during winter, summer, and rainy season at 26.2°C, 29.7°C, and 28.2°C, respectively [13]. Overall, the prevalence of SARS-CoV-2 infection has been relatively low compared to other countries, particularly during the earlier waves of the pandemic. To help facilitate wastewater surveillance in low prevalence areas, we developed and examined in the present study a quick and simple method for detecting SARS-CoV-2 in wastewater in Thailand, where the ambient temperature is high, and the prevalence of reported COVID-19 cases is relatively low during the early stages of the second outbreak wave.

## 2. Materials and Methods

The study was approved by the Institute Biosafety Committee (Protocol ID: MU 2021-002), and wastewater samples potentially containing SARS-CoV-2 were properly processed in accordance with standard biosafety guidelines from the World Health Organization [14].

### 2.1. Sample Collection

During January and February 2021, at the time of the second COVID-19 outbreak in Thailand, grab samples of wastewater were obtained from Chakri Naruebodindra Medical Institute (CNMI), a hospital treating patients with COVID-19 in Samut Prakan province, Thailand. All wastewater samples were collected downstream to the isolation ward for patients with confirmed COVID-19 and before the influent to a water treatment station. All samples were transported on ice to the laboratory for processing.

During February 2021, after social distancing and the closure of schools and specific crowded areas were implemented during the second wave of the SARS-CoV-2 pandemic in Thailand of which the epicenter was Samut Sakhon province (45 km southwest of Bangkok), sewage wastewater samples were collected from 46 open fresh markets in the Bangkok metropolitan area and in the Thanyaburi district of Pathum Thani province. The first confirmed COVID-19 case in Pathum Thani province, part of outer northern Bangkok, was found at the Pornpat market and officially reported on 22 December 2020. In the period from 4 to 13 January 2021, the SARS-CoV-2 infection rate rose to 1.31% (45 of 3,432 people from the Pornpat and Suchart markets tested positive for SARS-CoV-2 infection). The markets were closed during this outbreak but reopened on 16 January. The second COVID-19 outbreak started on 7 February, when another positive SARS-CoV-2 test result was reported. A period of “Active Case Finding” then began, spanning from 9 to 16 February 2021, during which 3,931 people at the Pornpat and Suchart markets were tested for SARS-CoV-2 infection. The infection rate rose to 8.37% (343 of 3,931). On 17 February, 14 waste water samples were collected from three local markets: Pornpat market, Suchart market, and Rangsit Market. During the sampling period, the Pornpat market and Suchart market had reported outbreaks, whereas the Rangsit market did not.

Pornpat market wastewater samples (P1–P4) were collected from the sewage pipeline network around the four corners of the market. Pornpat market samples were also collected from two water sampling sites (P50P6) in the Rangsit canal to check for contamination and from another two sites (P7-P8) at sewage pipelines from dwellings next to the Pornpat market. For the Suchart market, two wastewater samples (S1-S2) were collected from the sewage pipeline in the market, and another sample (S3) was collected from the Rangsit canal. For the Rangsit market, three waste water samples (R1–R3) were collected from the sewage pipeline network that passed through the middle of the market (Supplement Figure S1).

Grab samples of wastewater (500 mL/sample) were collected in clean plastic bottles from the wastewater drainage/sewage management system associated with each market and transported on ice to the laboratory, where they were stored at 4°C until use in further analysis.

### 2.2. Sample Preparation and Concentration

The sample preparation and concentrating method was adapted from the work of Ahmed et al. [15]. A subsample (100–400 mL) of each collected grab sample of wastewater was centrifuged at 3000 ×*g* for 10 min at room temperature to separate out the sediment. The resulting supernatant was then filtered by using a mixed cellulose ester membrane filter (pore size, 0.45 µm; diameter, 47 mm; GE Healthcare, Chicago, IL, USA) attached to a disposable Millicup™-FLEX filtration unit (Merck Ltd, Darmstadt, Germany), followed by applying the vacuum pump system to the assembly filtration unit until filtration was complete. Subsequently, the membrane filter was removed and placed in a sterile 5 mL tube. DNA/RNA Shield™ (1 mL) and 0.1 g of ZR BashingBead (Zymo Research, Sigma, Irvine, CA, USA) were added to each tube, after which the tubes were stored at −80°C until use in further RNA isolation.

### 2.3. RNA Extraction

To elute the viral RNA from the filtered mixed cellulose ester membrane, the preparation solution was first mixed 10 times (60 s each) with a vortex mixer at near maximum speed. After being mixed, 400 µL of the solution was transferred into a new nuclease-free tube. The viral RNA was then extracted by using the viral RNA mini kit (Qiagen, Hilden, Germany) in accordance with the manufacturer's protocol. The concentration and purity of the extracted RNA were determined by using a Nanodrop™ (Thermo Fisher Scientific, Waltham, MA, USA). The absorbance readings at 260 nm and 280 nm (260/A280 ratios) are commonly used to determine the purity of nucleic acid. A general acceptable range for 260/280 ratios is 1.9–2.1.

### 2.4. SARS-CoV-2 Detection and Quantification

For SARS-CoV-2 detection in wastewater samples, RT-qPCR assays were performed and analysed with a TaqMan™ 2019nCoV Assay Kit v1 (Thermo Fisher Scientific), which detects the following three SARS-CoV-2 viral genes: ORF1ab, spike (S), and nucleocapsid (N). Each 25 µL RT-PCR reaction mixture contained 6.25 *μ*L of 4x TaqPath™ 1-Step RT-qPCR Master Mix, 1.25 *μ*L of COVID-19 Real-Time PCR Assay Multiplex, 12.5 *μ*L of nuclease-free water, and 5 *μ*L of extracted RNA. TaqPath™ COVID-19 control (Thermo Fisher Scientific) was used as a positive control, MS2 Phage control was used as an internal positive control, and DNase/RNase-free water was used as a negative control. Each RT-qPCR experiment was performed on a ViiA 7 Real-Time PCR instrument (Applied Biosystems, Waltham, MA, USA) using the following thermocycling conditions: 2 min at 25°C for UNG incubation to eliminate amplicon carryover, 15 min at 50°C for reverse transcription, 2 min at 95°C for predenaturation, and 40 cycles of 3 s at 95°C and 30 s at 60°C for denaturation, annealing, and extension. After every amplification cycle, the fluorescence intensity was measured at 60°C. Results were classified as positive for SARS-CoV-2 detection if they included positive results, defined as cycle threshold (Ct) < 37, for two or more SARS-CoV-2 target genes. An individual assay result of 37 ≤ Ct ≤ 40 was considered to be inconclusive; samples with inconclusive results were repeated. A 10-fold serial dilution of TaqPath™ COVID-19 control amplification was performed to generate the standard curve used for SARS-CoV-2 quantification.

### 2.5. Sensitivity of SARS-CoV-2 Detection in Wastewater

To determine the lower limit of detection of the assay kit used in this study, we established a standard curve with 10-fold serial dilutions of 2019-nCoV DNA control from the RT-qPCR Kit, ranging from 1×10^4^–1 copies/µL. An inverse linear relationship was generated against each of the three target genes. The mean Ct values ranging from 26.9 ± 0.1 to 35.9 ± 1.1 for the ORF1ab gene, 26.7 ± 0.1 to 33.6 ± 0.4 for the N gene, and 26.1 ± 0.1 to 32.9 ± 0.6 for the S gene corresponded to concentrations of 10^4^–10^2^ copies/µL (Supplement Table S1).

For the sensitivity of the used method on grab wastewater, left-over grab wastewater samples negative for SARS-CoV-2 viral RNA stored at -80°c were pooled and mixed for analysis in a spike study. Seven hundred mL of the pooled samples was divided into 14 equal aliquots of 50 mL and processed in duplicate. Two aliquots of no-spike wastewater were autoclaved, to further ensure the absence of SARS-CoV-2 RNA in the samples, and were used as blank samples. Ten aliquots were individually spiked with 10-fold serial dilutions of inactivated culture medium of SARS-CoV-2 with 10% TRIzol™ reagent (cat. no. 15596026, Life Technologies Corporation, Carlsbad, CA 92008). Genomic copies (GC) number in all samples was determined by RT-qPCR. Each 50 mL spiked wastewater aliquot was then concentrated by the method adapted from the work of Ahmed et al. [15] before further RNA extraction and RT-qPCR as described in Materials and Methods.

## 3. Results

### 3.1. Sensitivity of SARS-CoV-2 Detection in Wastewater

Table 1 shows the results of the spike experiments with serially diluted virus culture medium samples into pooled wastewater samples negative for the virus. SARS-CoV-2 could be detected in 15/15 in all seeded samples with Ct values ranging from 18.28 ± 0.16 to 28.60 ± 0.41 for the N gene, 18.94 ± 0.06 to 29.13 ± 0.85 for the ORF1ab gene, and 19.37 ± 0.11 to 29.77 ± 1.54 for the S gene, respectively. Overall, the assay was sensitive enough to detect SARS-CoV-2 down to 1.63 ± 0.47, 1.20 ± 0.49, and 1.20 ± 1.51 copies/mL for the N, ORF1ab, and S genes, respectively. No virus was detected from all 4 samples extracted from no-spike pooled wastewater with or without being autoclaved before the extraction.

As shown in Table 2, the Ct values for the N, ORF1ab, and S genes progressively increased as the number of patients admitted to the treatment floor decreased. Notably, two out of the three SARS-CoV-2 genes were still detectable in wastewater even when only one patient was present in the COVID-19 isolation ward. The ORF1ab and S genes appeared to be more sensitive for detecting the presence of patients with COVID-19 from wastewater. As the number of patients with COVID-19 decreased, the Ct values for both these genes increased. There was a significant correlation between the number of cases and the Ct values of the ORF1ab gene (*r* = −0.99, *p* < 0.05), whereas such correlation for the S gene tended to reach statistical significance (*r* = −0.98, *p*=0.06). For the S genes, there were only 2 detectable samples, and the statistical analysis was not performed.

### 3.2. SARS-CoV-2 Surveillance in Wastewater from Fresh Markets in Bangkok

**Figure 1:**
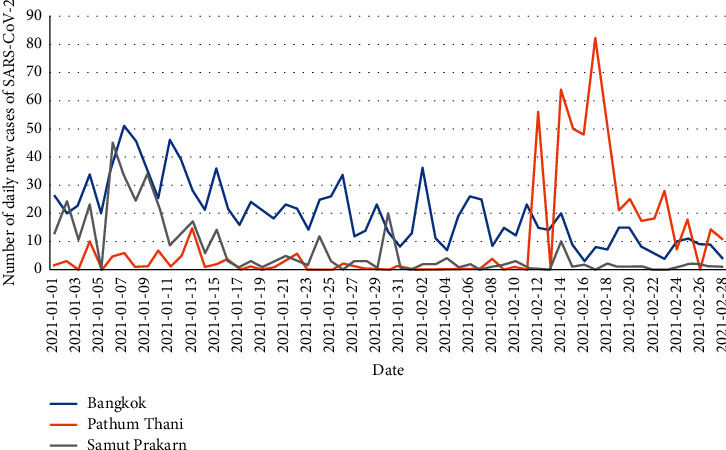
Number of daily new cases of SARS-CoV-2 infection in the Bangkok metropolitan (blue line), Pathum Thani province (orange line), and Samut Prakan province (grey line) from January to February 2021. Data obtained from the Department of Disease Control, Ministry of Public Health, Thailand [16].

Wastewater samples were collected from 46 open fresh markets in the Bangkok metropolitan area. The daily number of reported cases in Bangkok during this period is shown in Figure 1, and a map of the markets from which the wastewater samples were collected is in Supplement Figure S2. All samples collected and tested during this period were found to be negative for SARS-CoV-2. There was no major outbreak of SARS-CoV-2 infection in Bangkok for up to 1 month after the wastewater collection period.

### 3.3. SARS-CoV-2 Surveillance of Wastewater from Fresh Markets in Pathum Thani Province

The SARS-CoV-2 RNA detection results for these samples are presented in Table 3. SARS-CoV-2 RNA was detected in three of the four samples (P1–P2, P4) from the Pornpat market sewage pipeline network, but the fourth sample (P3) produced an inconclusive result. Only the ORF1ab gene was detected in all four samples from the Pornpat market sewage pipeline network (P1–P4); similarly, this gene was also the only one detected in both of the sewage pipeline samples from the Suchart market (S1-S2). Furthermore, SARS-CoV-2 RNA was detected in the samples from sewage pipelines from dwellings next to the Pornpat market (P7-P8). In contrast, SARS-CoV-2 genetic material was not detected in any samples from the Rangsit canal (P4-P5, S3) or the Rangsit market (R1–R3).

## 4. Discussion

In the present study, we demonstrated the performance of a quick and simple method for detecting SARS-CoV-2 in wastewater in Bangkok and the surrounding areas despite the high ambient temperature and low prevalence of COVID-19 in this location. Previous studies, such as those performed in the Netherlands [2] and Australia [1], have applied various methods of filtering, concentrating, and PCR to detect SARS-CoV-2 in sewage samples. Here, we chose to use membrane filtering and concentrating, which have been demonstrated as reliable methods for detecting SARS-CoV-2.

Recent studies in many countries identified SAR-CoV2 viral RNA in wastewater, sewage sludge, and river water using two or more genetic parts of the viral genome [17]. Three different SARS-CoV-2 genomic regions, Orf1ab gene, N gene, and S gene, were used to detect virus RNA in wastewater in Chile [18], India [12, 19], and Germany [20]. The studies revealed that the genome copy number of the viruses increased progressively, corresponding to an increase in the estimated number of virus infections in the community and the affected area. Furthermore, partly similar to our study, the Ct values have been demonstrated to negatively correlate to the effective reproduction number (Rt), daily COVID-19 hospitalization with a 33-day time delay, and daily changes in percent positivity among tested samples [21].

As for now, there are no assay targets recommended for identifying SARS-CoV-2 in wastewater. Several SARS-CoV-2 gene targets were commonly used for RT-qPCR detection such as N, E, S, RNA‐dependent RNA‐polymerase (RdRp, also known as nsp12), and open reading ORF1ab [22]. Bivins et al. reviewed the SARS-CoV2 RNA wastewater surveillance about the variability of basic and essential information for reverse transcription-quantitative PCR (RT-qPCR) assay parameters such as SARS-CoV-2 gene target, the standard curve parameters of *y*-intercept, slope and/or efficiency, and r2 value. They screened 208 RT-qPCR assays from 46 preprint and 36 peer-reviewed publications and found the assays targeting 130 N gene, 25 targeted ORF1, 23 targeted the E gene, 19 targeted RdRp, and 10 targeted the S gene, whilst one did not report any target molecule. Quantification SARS-CoV-2 RNA in wastewater targeting the US CDC N1 and N2 accounted for 45% of the RT-qPCR assays reported and N1 was tested more frequently (39%) than the US CDC N2 (32%) [23]. Therefore, we examined three different genes of SARS-CoV-2, ORF1ab, N protein genes and S protein genes, using the commercial PCR test kits, TaqMan™ 2019-nCoV Assay Kit v2 (Life Technologies Corporation, USA) for SARS-CoV-2 diagnosis of the virus genome in wastewater sample. This assay kit has been approved for marketing in Thailand by the Food and Drug Administration (FDA) Thailand under an evaluated for emergency use authorization (EUA) since June 4th, 2020 [24].

This study protocol allowed SARS-CoV-2 to be detected even when the number of apparently infected individuals in the wastewater catchment area was as low as one. Interestingly, although the RT-qPCR Ct values were related to both the absolute number and the percentage of persons infected with SARS-CoV-2 in the catchment area, the Ct values were more closely related to the absolute number of infected persons. This result is likely a consequence of the high sensitivity of RT-qPCR, which can detect SARS-CoV-2 RNA in samples with as little as 21 copies per reaction for the N1 gene [25]. Our findings also suggest that, at the building level, the amount of daily wastewater production may not only be related to the number of people using the sewage system.

Importantly, our study confirms that SARS-CoV-2 can be detected in wastewater from a number of areas in Thailand, where the ambient temperature is high and there is abundant sunshine all year round and the average temperature obtained from the Thai Meteorological Department [13] during collection of samples is 25.7 and 28.4 for January and February 2021, respectively. The average daily maximum UV index is 10 in January and up to 12 in February.

Our finding is in line with a report from India, where the weather is relatively similar to that of Thailand; SARS-CoV-2 could be detected in wastewater from India during the early phase of the SARS-CoV-2 pandemic [12]. In that study, water was sampled from a water treatment plant in Ahmedabad, for which the catchment areas included a hospital that was treating patients with COVID-19. Ahmedabad is located close to the equator and has a high ambient temperature, averaging 27.1°C [26]. The number of COVID-19 cases in Ahmedabad during the course of that study was approximately 5,000–10,000, which is much higher than the COVID-19 prevalence in Thailand during the present study. A few other countries in Asia have also reported the successful detection of SARS-CoV-2 in wastewater. In Japan, during March to May 2020, wastewater samples were collected from several water treatment plants, and the SARS-CoV-2 detection frequency was found to increase with the number of reported COVID-19 cases [27]. Interestingly, SARS-CoV-2 could be detected even when the number of COVID-19 cases was <1.0 per 100,000 people. Another study with sample sources and a setting similar to ours identified SARS-CoV-2 in wastewater from a hospital in China [28], although unlike our study, the number of COVID-19 cases in China at the time of that study was very high. Our study is in line with a report from Wannigama et al. [29], who reported the detectability of SARS-CoV-2 in wastewater in Bangkok and suggested that wastewater can be used as a complementary source for detecting the viral RNA and predicting upcoming outbreaks. They monitor SARS-CoV-2 RNA in wastewater prior to the second outbreak in Thailand during the rainy season and winter.

In the present study, RT-qPCR performed using a TaqMan™ 2019-nCoV Assay Kit v2 was used to detect three SARS-CoV-2 target genes: ORF1ab, S, and N. Our results demonstrate that the ORF1ab gene was detected at a higher frequency compared with the N and S genes. Overall, eight out of eight (100%) ORF1ab gene assays produced positive amplifications, whereas only five out of eight (62.5%) assays for the N and S genes did so; the average Ct values were 32.95 ± 2.06, 34.04 ± 1.53, and 34.21 ± 2.03 for the ORF1ab, N, and S genes, respectively. A previous study of this RT-qPCR kit reported differences in its sensitivity for detecting different SARS-CoV-2 target genes in nasopharyngeal swabs from 98 COVID-19-positive patients. The highest detection sensitivity was for the N gene (76.5%; 95% confidence interval: 66.9%–84.5%), followed by the S gene (70.4%), and the ORF1ab gene (65.3%) [30]. Using the RNA-dependent RNA polymerase (RdRP), N, and S genes as targets, another study on wastewater samples found that the amplification efficiencies were 93%, 87%, and 84%, respectively [31]. The sensitivity differences among gene targets and different studies have been suggested to reflect differences in the abundance of SARS-CoV-2 in wastewater according to the community COVID-19 pandemic level and the methodologies applied for viral RNA detection, including those for virus concentration, RNA extraction, and RT-qPCR assay [32].

## 5. Conclusions

- The membrane filtering-based method described here is a rapid, extremely simple, and sensitive approach for the detection of SARS-CoV-2 in wastewater from areas with low numbers of COVID-19 cases
- Wastewater monitoring for SARS-CoV-2 is sensitive and can detect the virus even in places with a high ambient temperature and relatively low prevalence of COVID-19
- This data is useful for community SARS-CoV-2 surveillance and prevention of the spread of coronavirus disease or COVID-19

## Figures and Tables

**Table 1:** SARS-CoV-2 detection from pooled wastewater negative for SARS-CoV-2 with and without SARS-CoV-2 spike.

SARS-CoV-2 seeded (GC)	N		ORF1ab		S	
	Mean Ct + SD	Copies/mL ± SD	Mean Ct + SD	Copies/mL ± SD	Mean Ct + SD	Copies/mL ± SD
2 × 10e4	18.28 ± 0.16	4,821.14 + 1,250.18	18.94 ± 0.06	5,081.59 + 1,785.77	19.37 ± 0.11	9,031.87 + 4,093.59
2 × 10e3	19.85 ± 0.08	1,145.74 + 58.11	20.04 ± 0.42	546.70 + 7.08	19.65 ± 0.13	893.20 + 12.93
2 × 10e2	22.33 ± 0.10	114.03 + 7.79	23.29 ± 0.16	86.57 + 3.94	22.54 ± 0.24	92.21 + 0.82
2 × 10e1	24.39 ± 0.08	12.07 + 1.51	27.44 ± 2.68	11.72 + 15.74	26.79 ± 2.69	11.89 + 17.23
2 × 10e0	28.60 ± 0.41	1.63 + 0.47	29.13 ± 0.85	1.20 + 0.49	29.77 ± 1.54	1.63 + 1.51
No-spike^a^	UD	—	UD	—	UD	—
No-spike^b^	UD	—	UD	—	UD	—

^a^Pooled wastewater where SARS-CoV-2 viral RNA was absent. ^b^Autoclaved pooled wastewater where SARS-CoV-2 viral RNA was absent. GC: genomic copies. UD: undetermined.

**Table 2:** Detection of SARS-CoV-2 in wastewater from a hospital where patients with COVID-19 were treated.

Date	Number of admitted patients	Number of estimated people in the wastewater catchment	Ct value		
			N	ORF1ab	S
12 Jan 2021	18	866	26.51	27.25	27.63
19 Jan 2021	4	839	31.94	33.89	32.01
26 Jan 2021	3	847	UD	33.43	33.65
27 Jan 2021	1	848	UD	35.74	36.51
28 Jan 2021	0	842	UD	UD	UD

UD: undetermined.

**Table 3:** Detectability of SARS-CoV-2 in samples from various markets in Pathum Thani province.

Location		Ct value			
		N	ORF1ab	S	Result
*Pornpat market*	P1	34.21	31.89	33.98	Positive
	P2	32.24	30.94	34.85	Positive
	P3	UD	33.35	UD	Inconclusive
	P4	UD	33.83	36.97	Positive
	P5	UD	UD	UD	Negative
	P6	UD	UD	UD	Negative
	P7	33.39	30.46	31.32	Positive
	P8	33.96	31.77	33.92	positive

*Suchart market*	S1	UD	36.51	UD	Inconclusive
	S2	36.41	34.81	UD	Positive
	S3	UD	UD	UD	Negative

*Rangsit market*	R1	UD	UD	UD	Negative
	R2	UD	UD	UD	Negative
	R3	UD	UD	UD	Negative

UD: undetermined.

## Data Availability

The data used to support the finding of this study are included within the article.

## Supplementary Materials

Supplementary Materials
*Table S1.* Quantification of a tenfold serial dilution of 2019-nCoV DNA control by real-time qPCR for the lower limit of detection assay. *Figure S1*. Location of markets in Pathum Thani from which wastewater samples were collected. The  symbol represents sites that tested negative, the  symbol represents sites that tested inconclusive, and the  symbol represents sites that tested positive for SARS-CoV-2 RNA. *Figure S2.* Location of the markets from which wastewater samples were collected in Bangkok
