# Supplementary material for: A Simple Method to Detect SARS-CoV-2 in Wastewater at Low Virus Concentration
Source: J Environ Public Health. 2022 Feb 22;2022:4867626. doi: 10.1155/2022/4867626 (PMC8888108; doi:10.1155/2022/4867626)
Supplement: Supplementary Materials — Table S1. Quantification of a tenfold serial dilution of 2019-nCoV DNA control by real-time qPCR for the lower limit of detection assay. Figure S1. Location of markets in Pathum Thani from which wastewater samples were collected. The symbol represents sites that tested negative, the symbol represents sites that tested inconclusive, and the symbol represents sites that tested positive for SARS-CoV-2 RNA. Figure S2. Location of the markets from which wastewater samples were collected in Bangkok [file 4867626.f1.docx]

**Supplements**

**Table S1.** Quantification of a ten-fold serial dilution of 2019-nCoV DNA control by real-time qPCR for the lower limit of detection assay

| Standard /concentration (copies/µL) |  | | Ct value | |  | |
| --- | --- | --- | --- | --- | --- | --- |
|  | N | | ORF1ab | | S | |
|  | mean Ct | SD | mean Ct | SD | mean Ct | SD |
| 10000 | 26.7 | 0.1 | 26.9 | 0.1 | 26.1 | 0.1 |
| 1000 | 29.9 | 0.1 | 30.3 | 0.2 | 29.3 | 0.2 |
| **100** | **33.6** | **0.4** | **35.9** | **1.1** | **32.9** | **0.6** |
| 10 | 37.9 | 0.2 | UD | - | 39 | 0.1 |
| 1 | UD | - | UD | - | UD | - |

SD, standard deviation; UD, undetermined

**
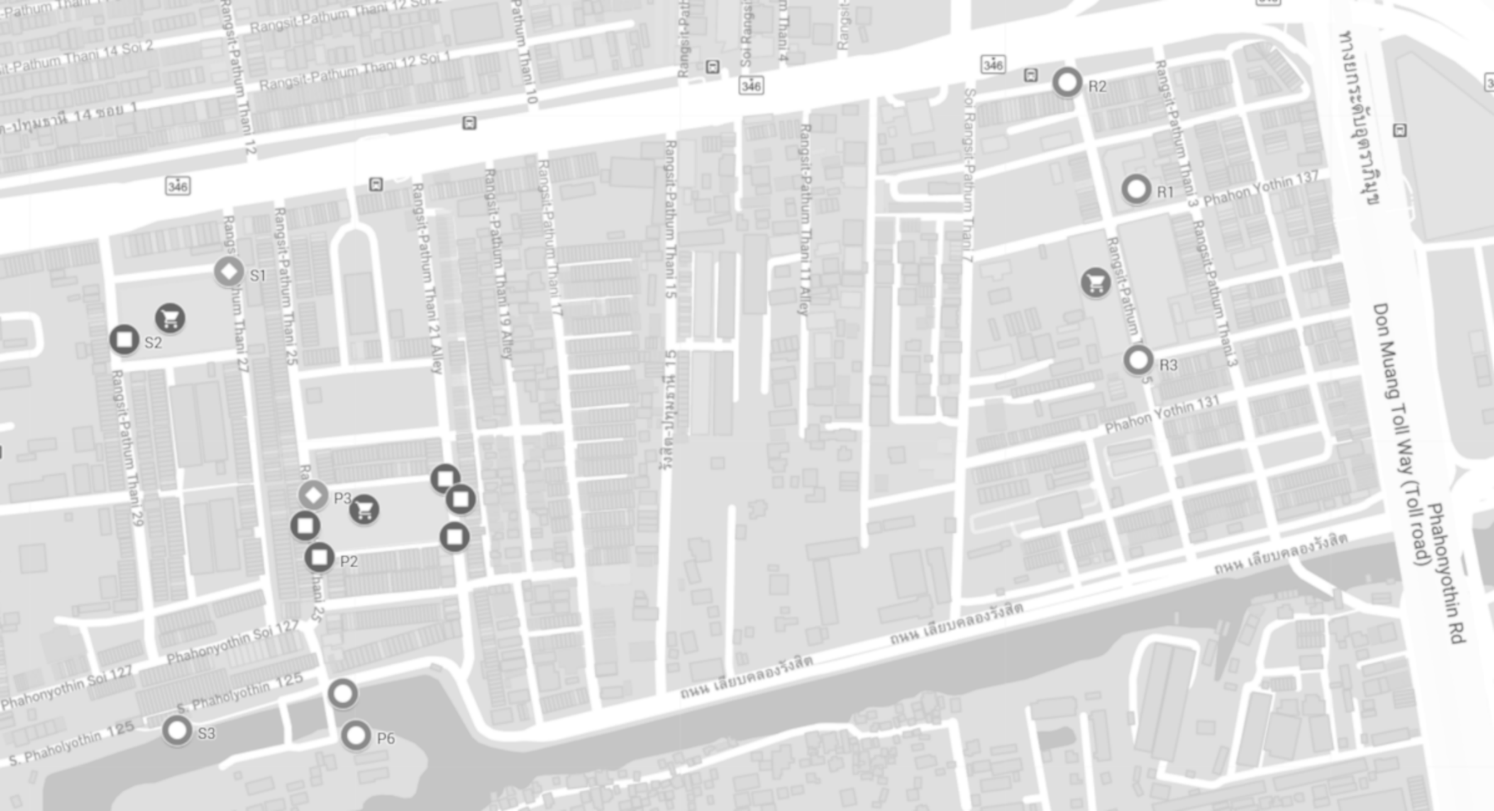
Figure S1** Location of markets in Pathum Thani from which wastewater samples were collected. The
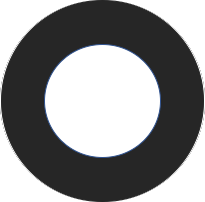
 symbol represents sites that tested negative, the
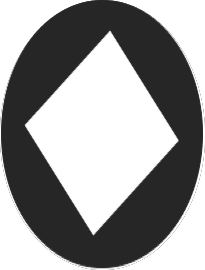
 symbol represents sites that tested inconclusive, and the
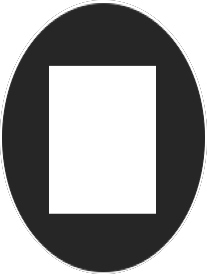
 symbol represents sites that tested positive for SARS-CoV-2 RNA.

**
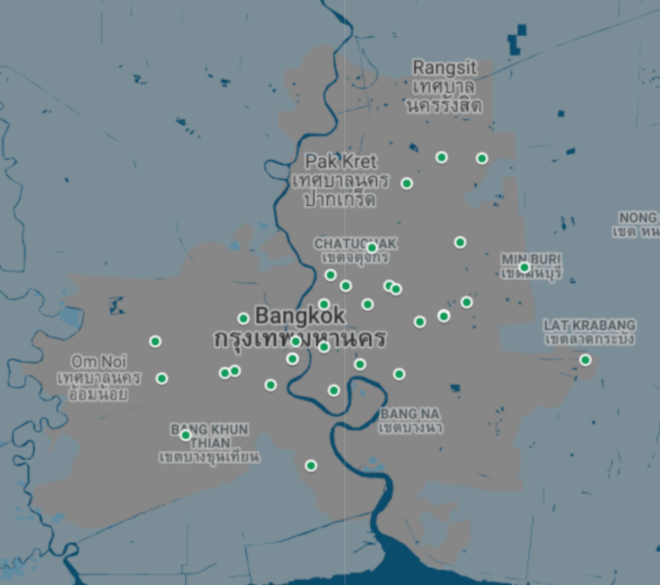
**

**Figure S2** Location of the markets from which wastewater samples were collected in Bangkok.
